# Supplementary material for: Circadian control of lung inflammation in influenza infection
Source: Nat Commun. 2019 Sep 11;10:4107. doi: 10.1038/s41467-019-11400-9 (PMC6739310; doi:10.1038/s41467-019-11400-9)
Supplement: Supplementary file 1 — Supplementary Information [file 41467_2019_11400_MOESM1_ESM.pdf]

Circadian control of lung inflammation in influenza infection

Corresponding author: Shaon Sengupta

Supplementary Figure 1

Score	Description
1	Percolated fur but no detectable behavior differences from untreated control mice
2	Mice with percolated fur and a huddle reflex but respond to stimuli (such as a tap on their cage) appropriately and are just as active upon handling as untreated control mice
3	Exhibit a slower response to a tap on the cage and that were passive or docile when handled but still curious when alone in a new setting
4	Exhibit lack of curiosity and little response to stimuli and that appear quite immobile.
5	Exhibit labored breathing and are unable or slow to right themselves after being rolled onto their backs (moribund)
6	Dead mouse.

Supplementary Figure 1: Scoring system for mice infected with influenza (1-6)

Supplementary Figure 2

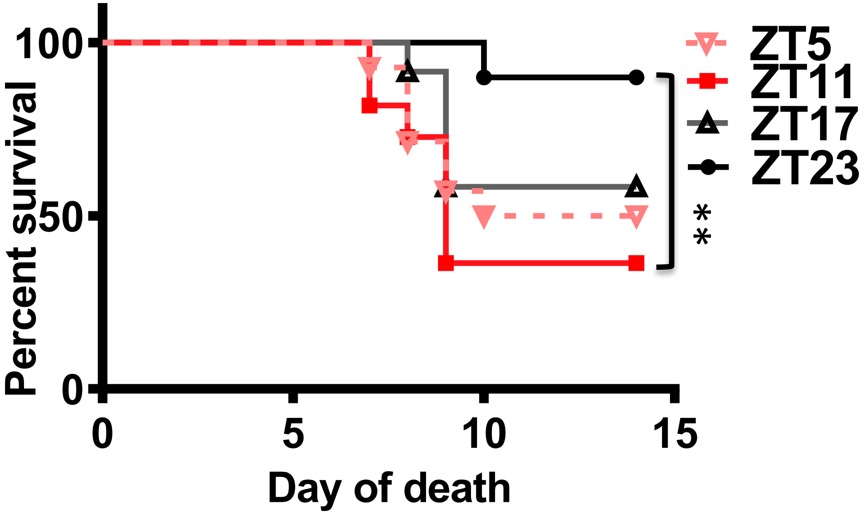

Supplementary Figure 2: Survival from IAV across circadian day: Four groups of mice were maintained under 12 hrs Light: Dark cycles. Mice were infected at ZT5, ZT11, ZT17 and ZT23 and were weighed at serial time points following infection as in Figure 1. Survival curves are a composite of 2 independent experiments [total n = 13-17 per group, logrank(Mantel–Cox) test, $p < 0.0001$].

Supplementary Figure 3

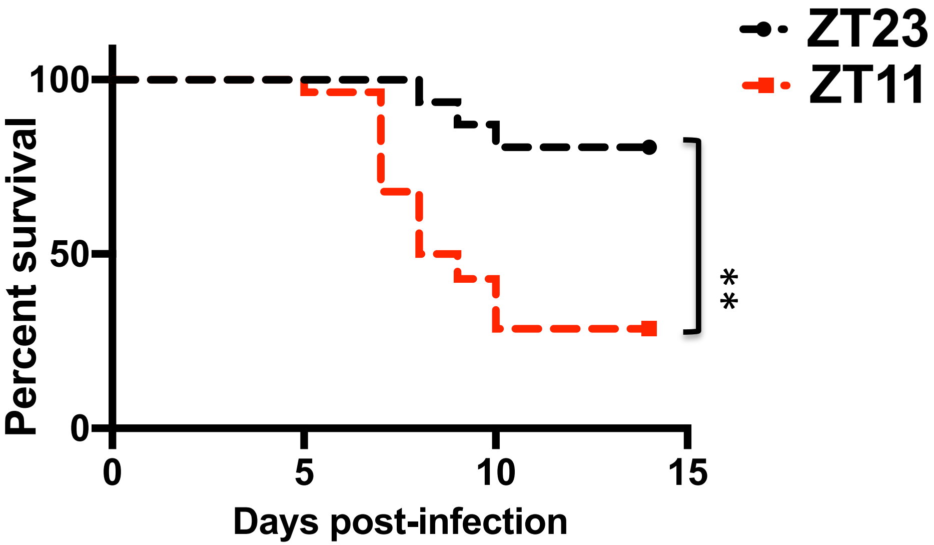

Supplementary Figure 3: Survival from IAV in reverse light-dark cycles: Two groups of mice were maintained in 12 hrs Light: Dark cycles and then placed under reverse light dark cycle at 8 weeks of age in specially designed circadian boxes. They were acclimatized to this schedule for ZT 23 or ZT 11; thereafter followed at serial intervals of time as in Figure 1. Survival curves are a composite of 3 independent experiments [total n = 17-20 per group, log-rank (Mantel-Cox) test, $p < 0.0001$].

Supplementary Figure 4

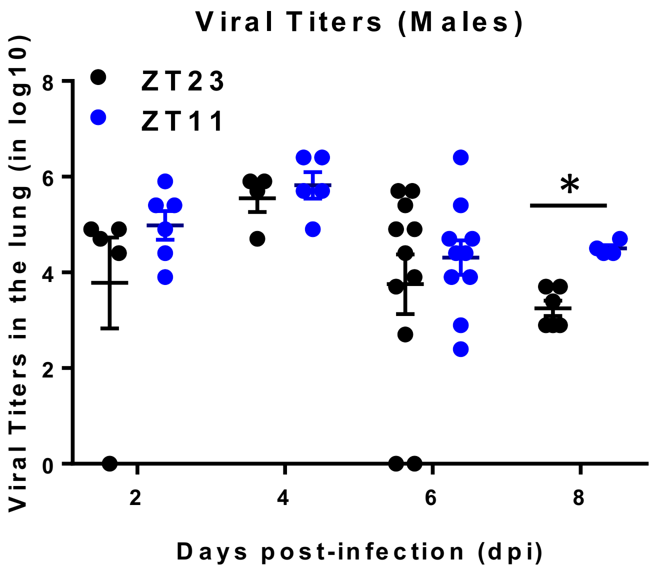

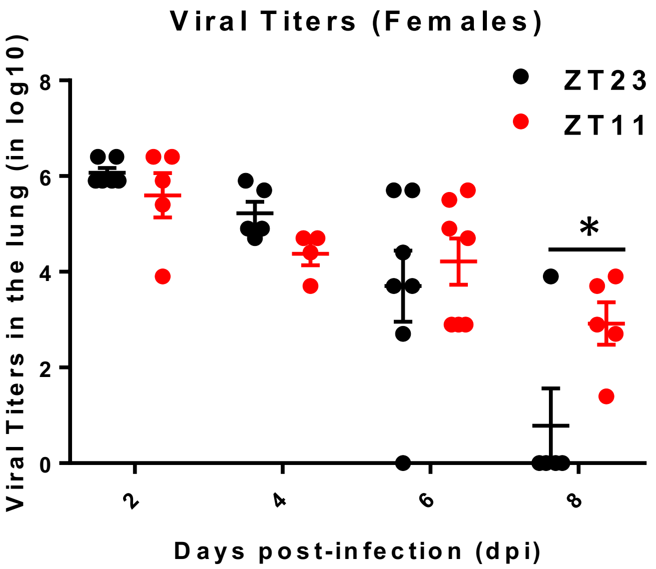

Supplementary Figure 4: Viral Titers stratified by gender. Data from viral titers segregated by gender as in Figure 2. (n=5-10/group; student t test; *p < 0.05, ZT23 vs. ZT11; Data was pooled across 3-4 independent experiments and is reported as mean ± SEM)

Supplementary Figure 5

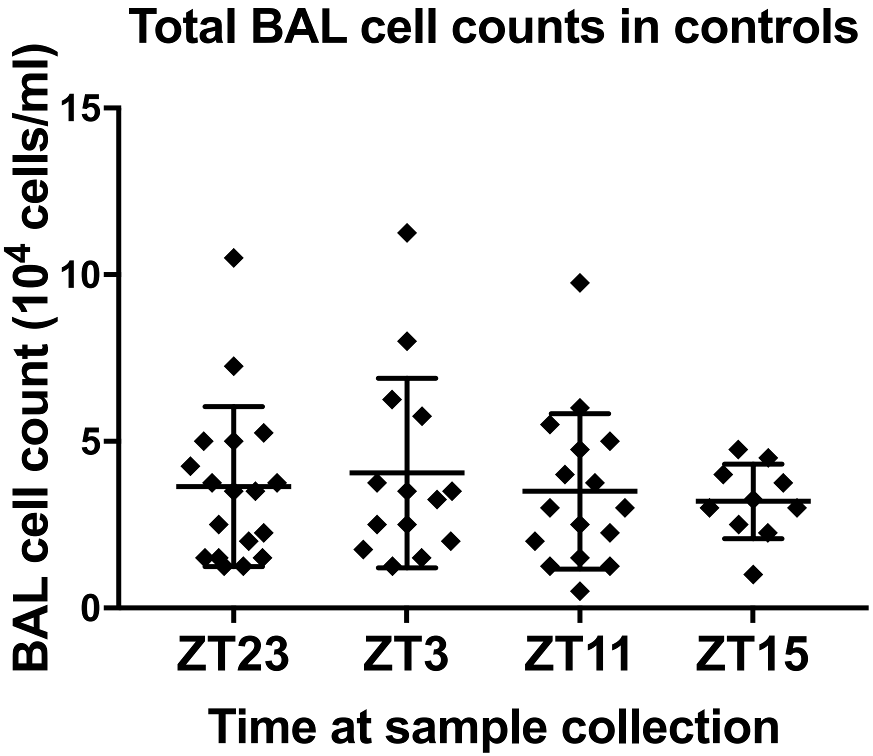

Supplementary Figure 5: BAL cell counts in control animals. BAL was collected as in Figure 3, at the indicated time points post-infection from mice that received PBS. Data compiled from 6 independent experiments are expressed as mean ± SEM (total n = 10-17 per time point)

Supplementary Figure 6

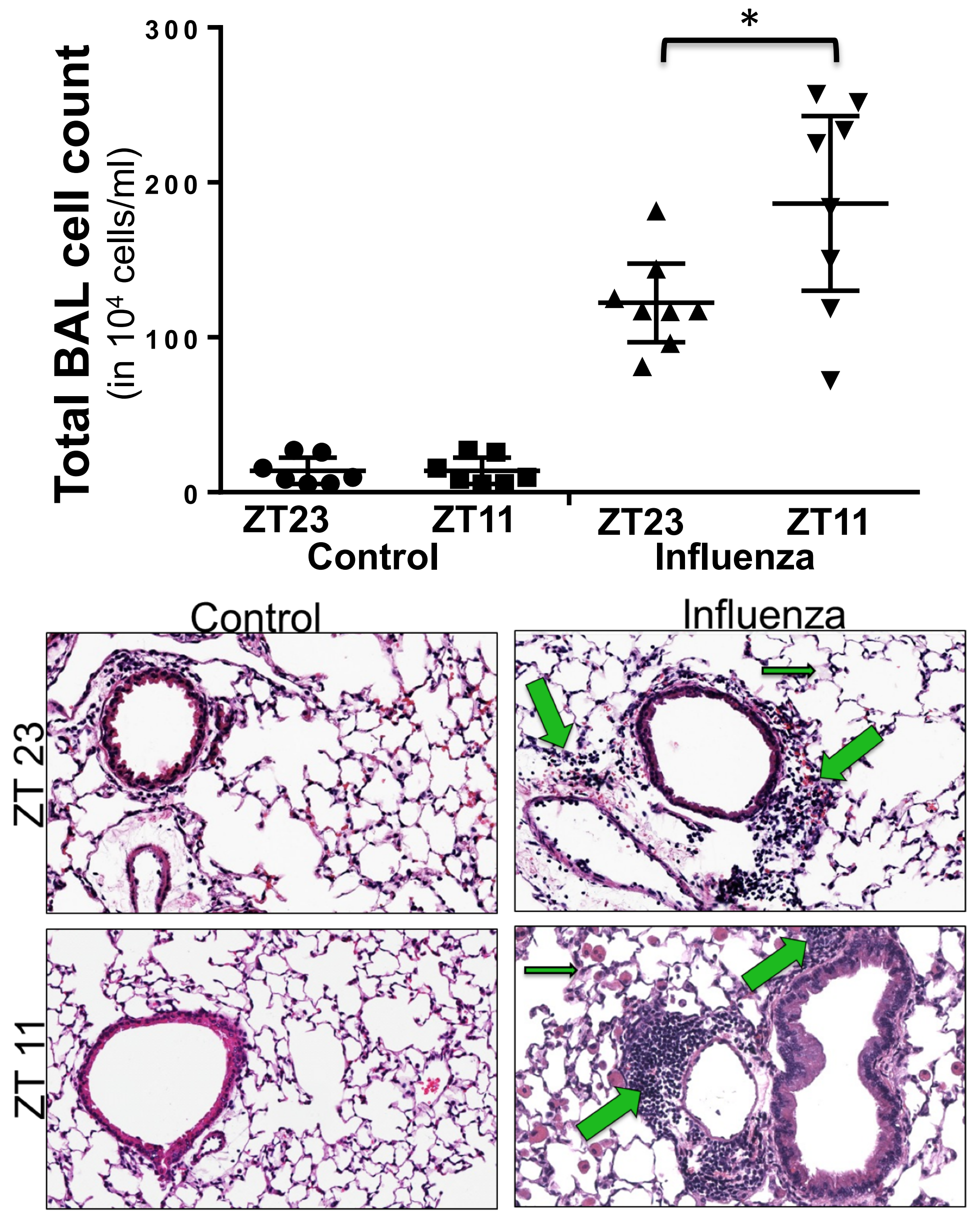

Supplementary Figure 6: Inflammation in X31 infected animals: Total BAL cell count on day 6 p.i. from mice who received either IAV (X31 strain) or PBS at either ZT23 and ZT11. Data compiled from 3 independent experiments are expressed as mean ± SEM (total n = 8 per time point, two-way ANOVA; *$p < 0.05$ ZT23 vs. ZT11).*(Lower panel*) Representative micrographs of H&E stained lung sections 5 days after sham intranasal inoculation (PBS) or IAV (strain X31) treatment.

Supplementary Figure 7

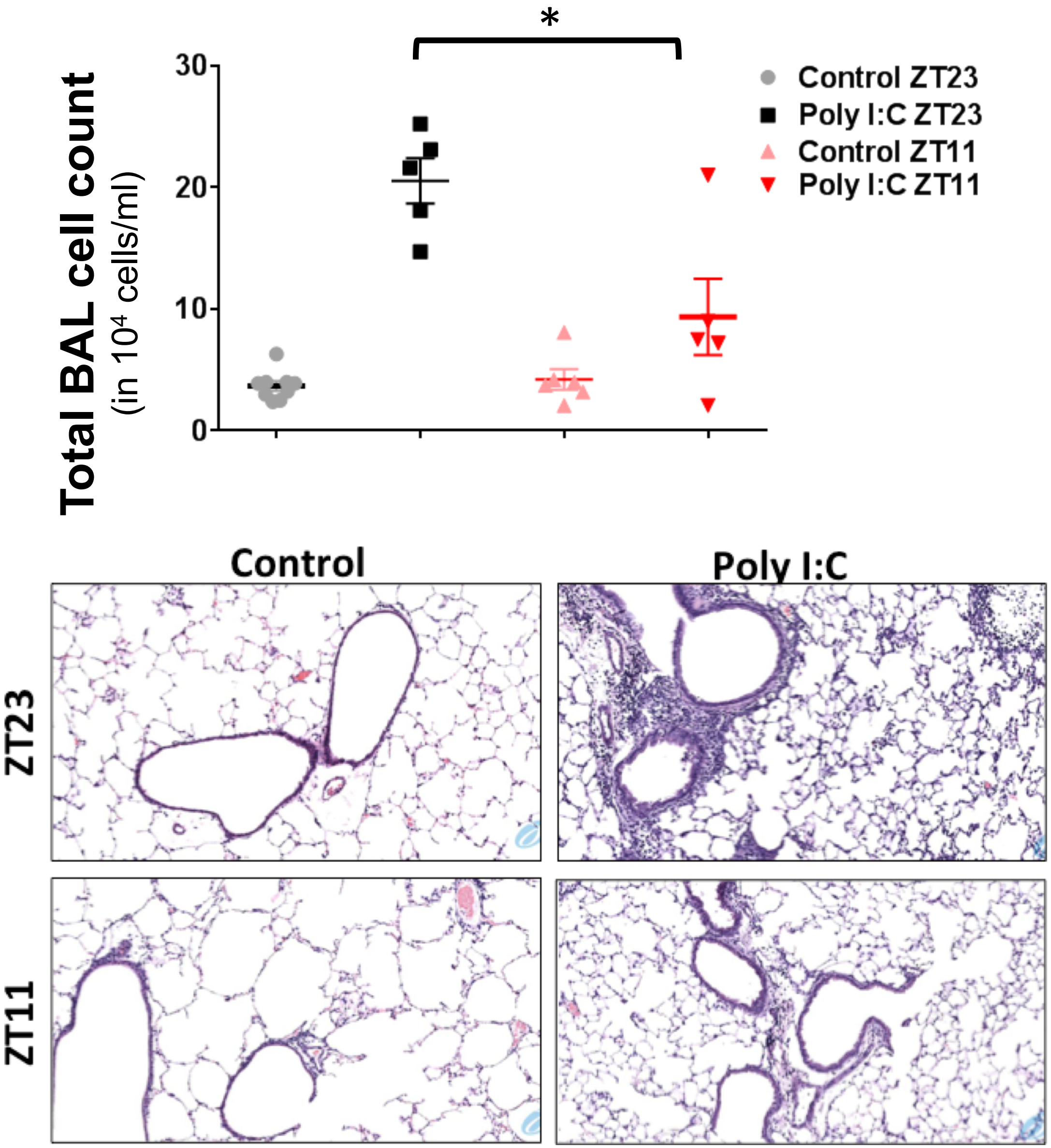

Supplementary Figure 7: Total BAL cell counts 24 hrs after administration of i.n. PBS or PolyI:C (*top panel*). Representative histology of lungs from the experiments above. (*lower panel*)

Supplementary Figure 8

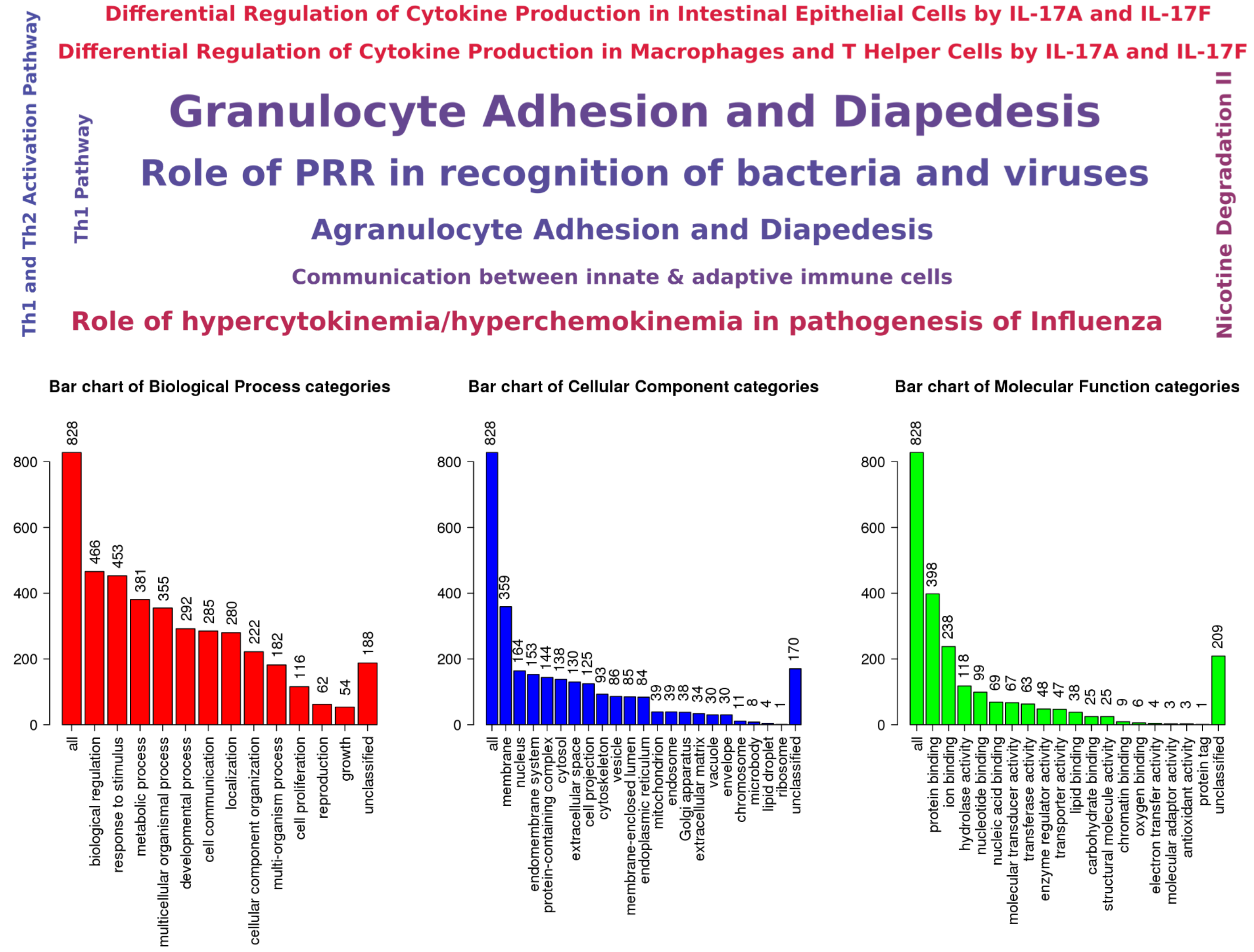

Supplementary Figure 8: (top panel) Word cloud representation of the IPA analyses from the transcriptomic profile from Fig 4. (lower panel): Bar chart if biological processes, cellular components involved and molecular functions affected, based on the differentially expressed genes.

Supplementary Figure 9

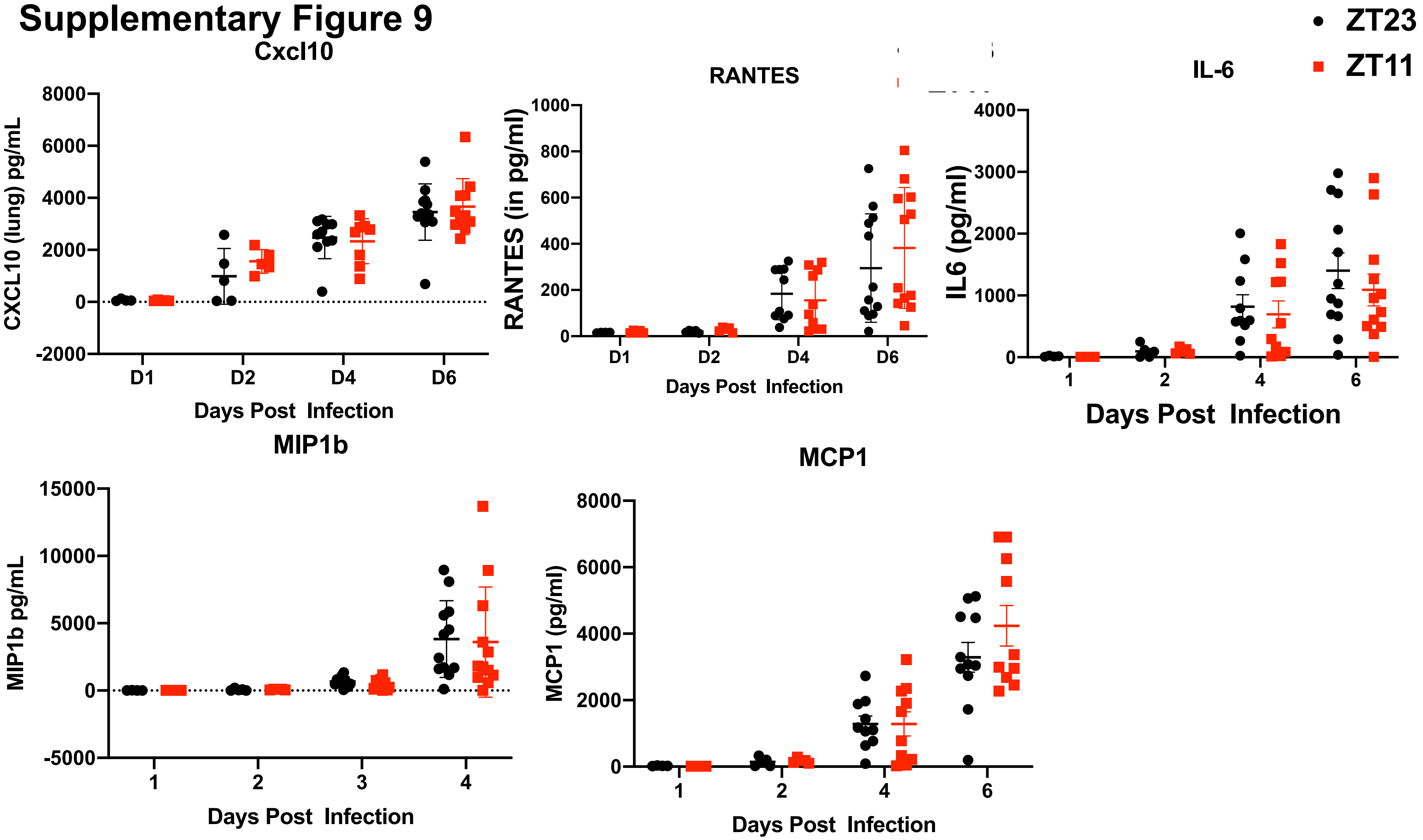

Supplementary Figure 9: Cytokine analyses from the supernatants of lung homogenates on days post-infection. (n=4-11/group; pooled data from 3 independent experiments).

Supplementary Figure 10(A)

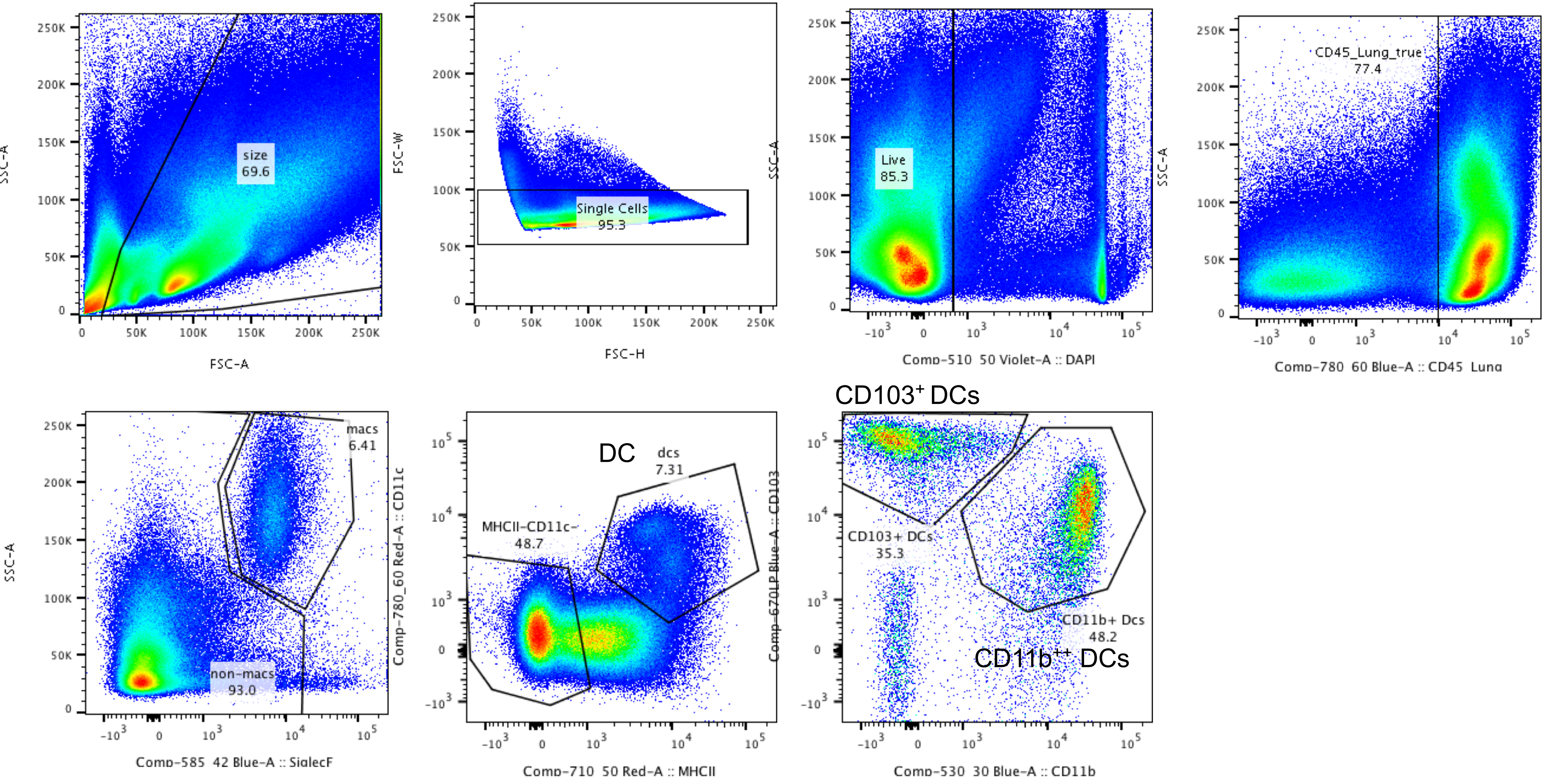

Supplementary figure 10(A): Gating strategy (DCs and macrophages).

Supplementary Figure 10(B)

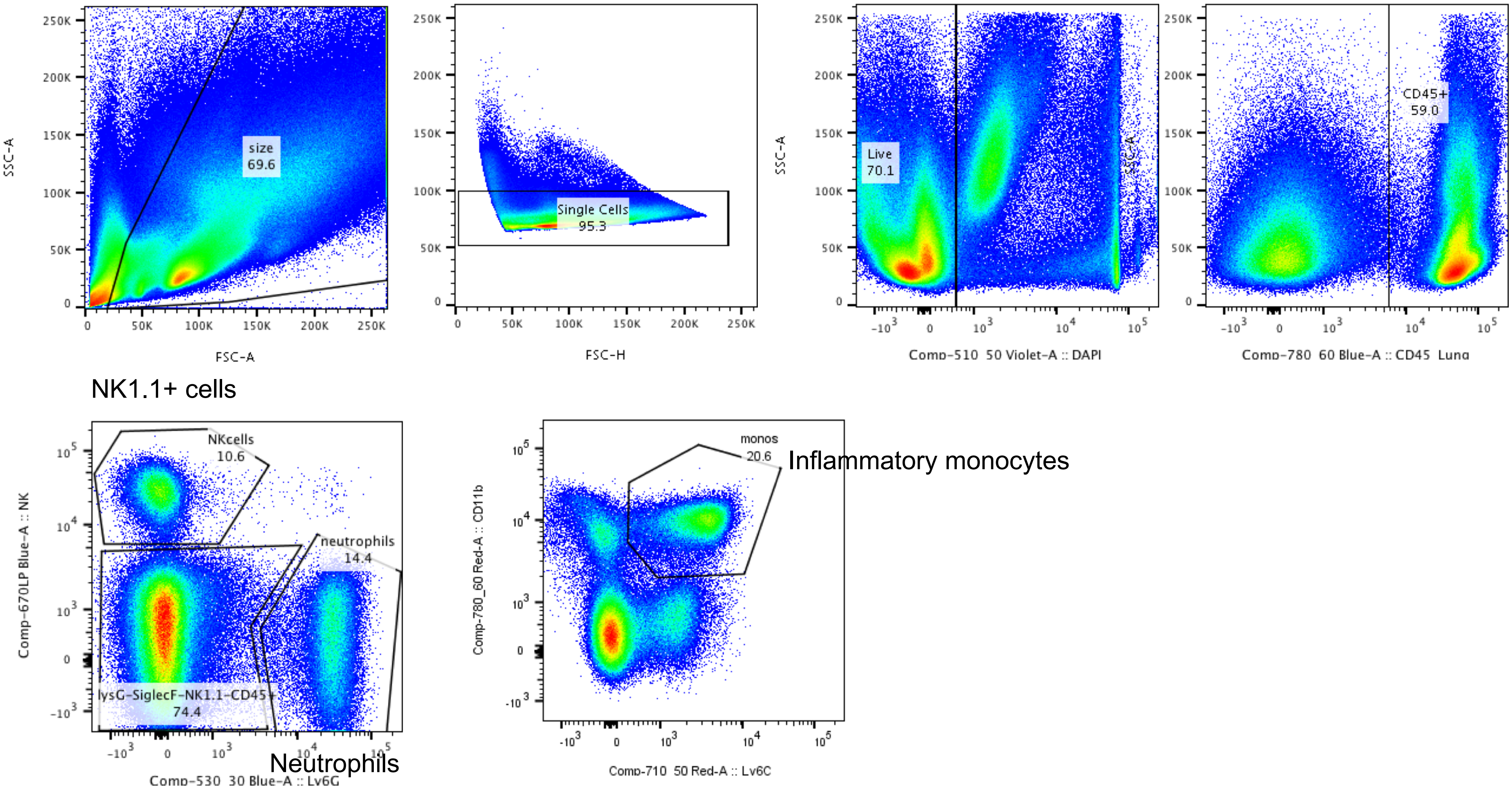

Supplementary figure 10(B): Gating strategy (neutrophils, NK1.1+ cells, inflammatory monocytes).

Supplementary Figure 11(A)

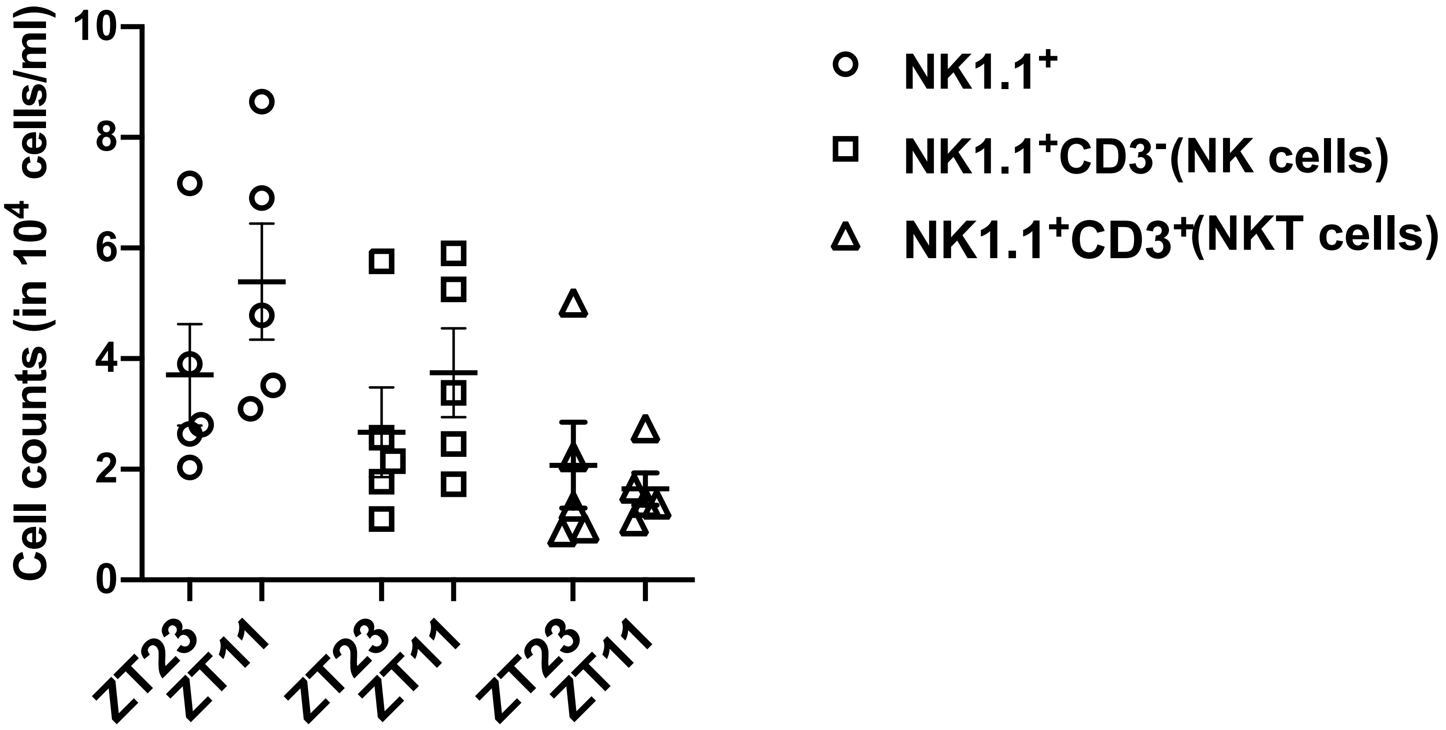

Supplementary Figure 11(A): Absolute NK1.1+ cell counts in uninfected lungs at ZT11 and ZT23.
(n=5/group; results on one experiment shown here)

Supplementary Figure 11(B)

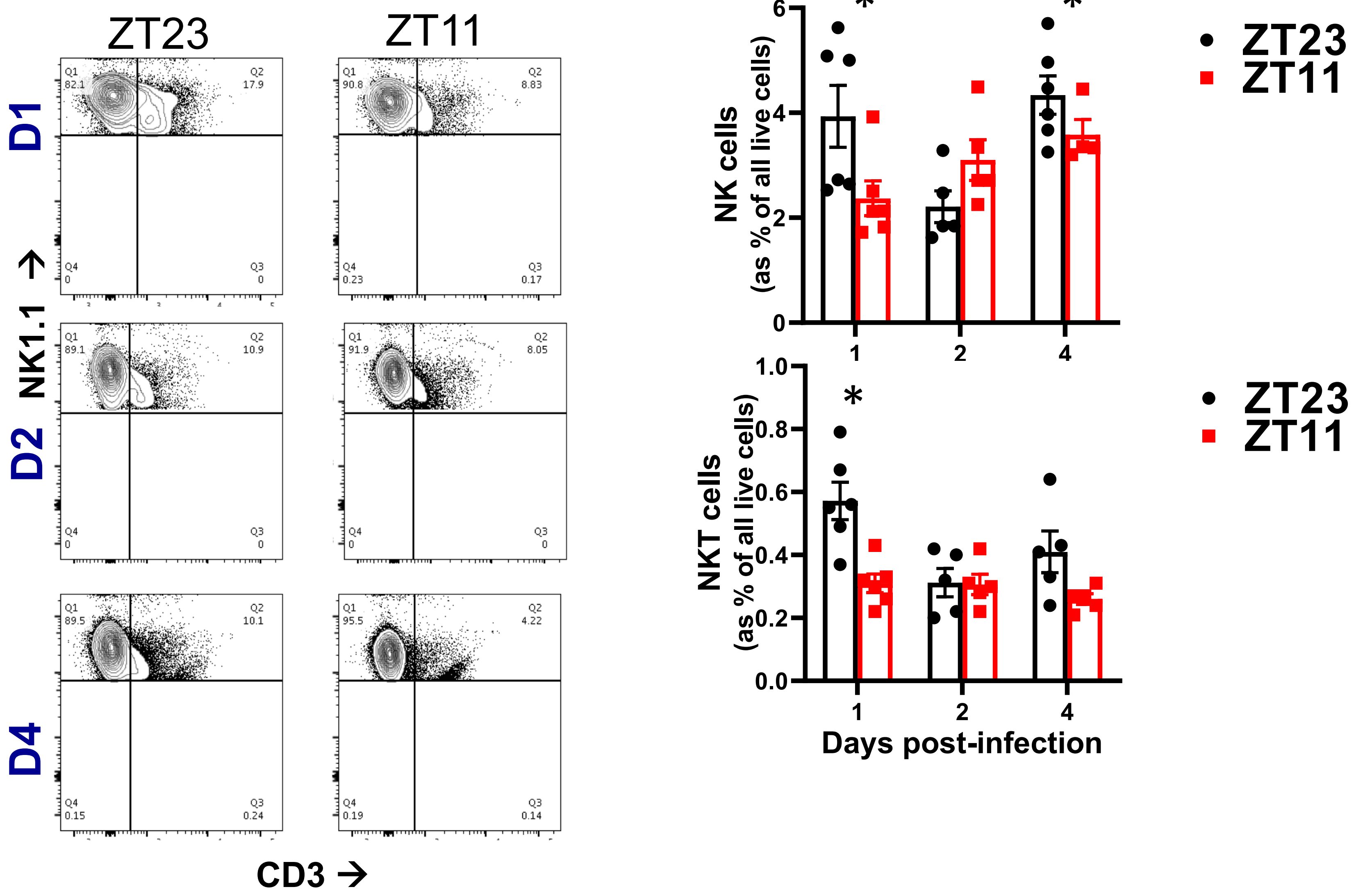

Supplementary Figure 11(B): NK cells and NKT cells. For NK cells 2-way ANOVA, p=ns (0.17) for time of infection and $p<0.05$ for day of dissection and $p<0.05$ for interaction. For NKT cells 2-way ANOVA, $p<0.001$ for time of infection and $p<0.05$. for day of dissection and p=0.03 interaction. (N=4-7/group per time point. Two-way ANOVA). Representative results from one experiment. Experiment repeated with similar results 3-5 times.

Supplementary Figure 12

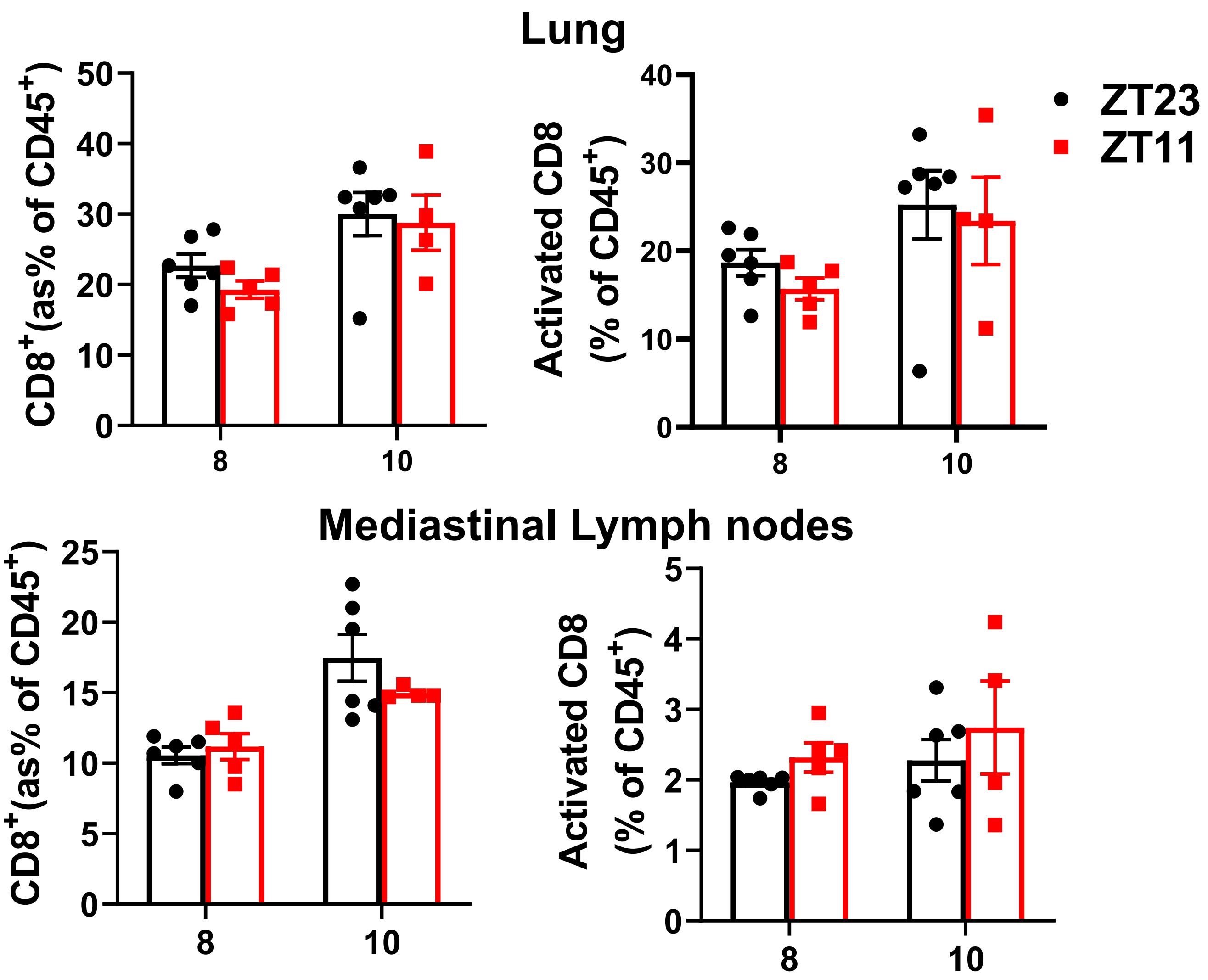

Supplementary Figure 12: CD8+ cells and activated CD8+ cells as a percentage of all CD45+ cells in the lung (*top panel*) and mediastinal infection (*lower panel*)

Supplementary Figure 13

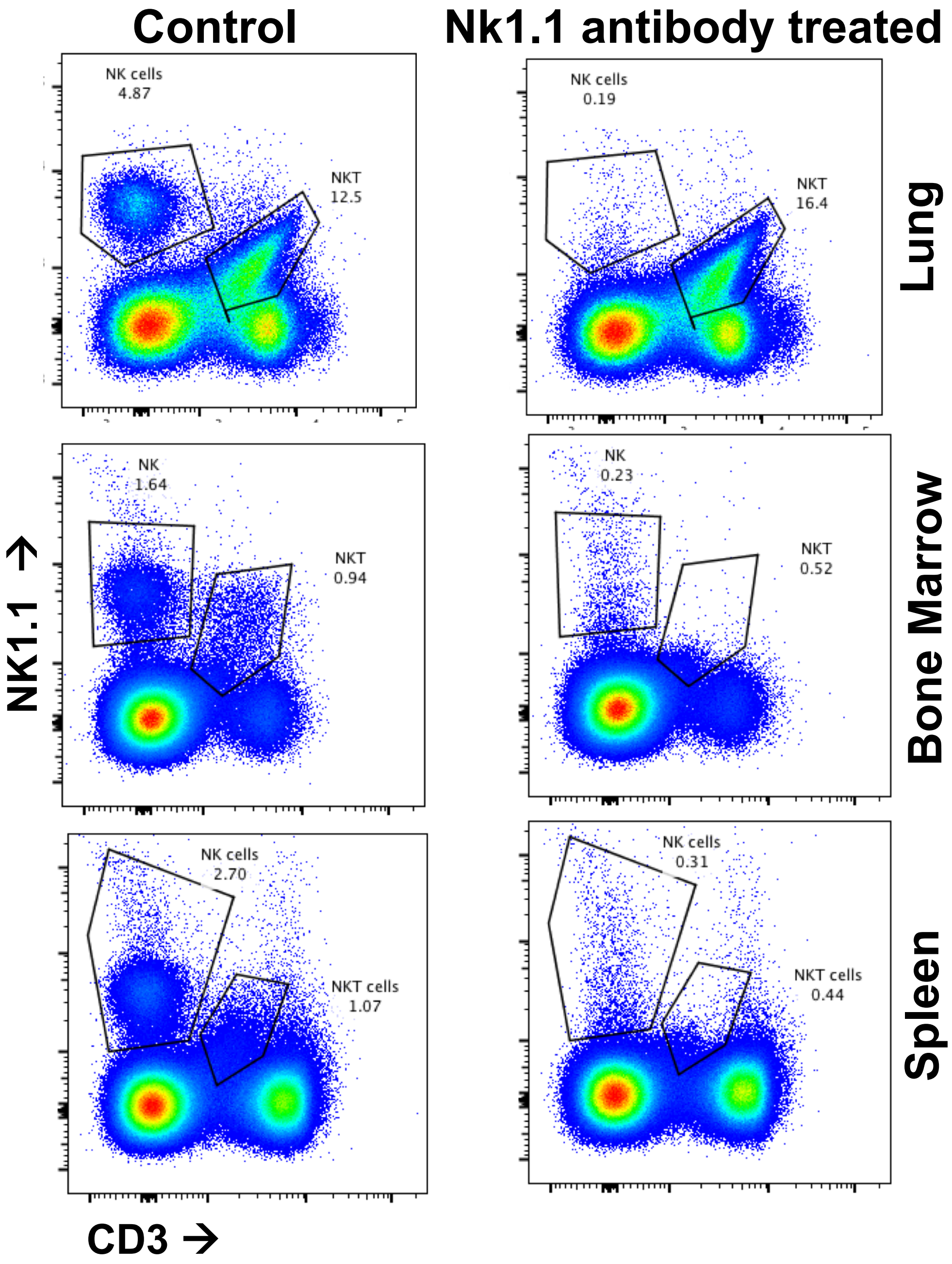

Supplementary Figure 13: Demonstration of depletion of Nk cells by NK1.1 antibody by flowcytometry (24hrs following antibody/mock injection).

Supplementary table 1

Antibodies used

Antibody	Clone	Company	Fluorophore	catalog number	lot number	Dilution
MHCII	M5/114.15.2	eBioscience	AF700	56-5321-82	1919519	1:100
NK1.1	PK136	Biolegend	PerCP-Cy5	108716	B242312	1:100
Ly6C	HK1.4	Biolegend	AF700	128024	B243043	1:100
CD11c	N418	Biolegend	APCCy7	117324	B237079	1:100
CD8	53-6.7	Biolegend	APCCy7	100714	B237526	1:100
Ly-6G	1A8	Biolegend	FITC, Pe	127606, 127608	B261239, B258704	1:100
CD11b	M1/70	eBioscience	FITC, APC cy7	11-0112-82, 47-0112-82	4341634, 1950123	1:100
CD4	GK1.5	Biolegend	FITC	100406	B245891	1:100
CD3	17A2	eBioscience	FITC	11-0032-82	1959695	1:50
Ly-6G	1A8	Biolegend	PB, FITC, PE	127606, 127608	B261239, B258704	1:100
CD45	30-F11	Biolegend	PE/Cy7	103114	B243728, B243728, B271123	1:100
CD4	GK1.5	Biolegend	FITC	100406	B245891	1:100
SiglecF	E50-2440	BD Pharmingen	PE	552126	7208832	1:100
CD62L	MEL-14	Biolegend	PE	104407	B242685	1:100
CD103	2E7	eBioscience	PerCP-710	46-1031-82	4339659	1:100
CD44	IM7	eBioscience	PerCP-Cy5.5	45-0441-82	4329935	1:100
Fcblock	93	Biolegend	N/A	101320	b218499	1:100
