# Supplementary material for: Circadian control of lung inflammation in influenza infection
Source: Nat Commun. 2019 Sep 11;10:4107. doi: 10.1038/s41467-019-11400-9 (PMC6739310; doi:10.1038/s41467-019-11400-9)
Supplement: Supplementary file 3 — Reporting Summary [file 41467_2019_11400_MOESM3_ESM.pdf]

nature research

Corresponding author(s): *Double-blind peer review submissions: write DBPR and your manuscript number here instead of author names.*

Last updated by author(s): *YYYY-MM-DD*

Reporting Summary

Nature Research wishes to improve the reproducibility of the work that we publish. This form provides structure for consistency and transparency in reporting. For further information on Nature Research policies, see Authors & Referees and the Editorial Policy Checklist.

Statistics

For all statistical analyses, confirm that the following items are present in the figure legend, table legend, main text, or Methods section.

n/a	Confirmed	
☐	☒	The exact sample size (*n*) for each experimental group/condition, given as a discrete number and unit of measurement
☐	☒	A statement on whether measurements were taken from distinct samples or whether the same sample was measured repeatedly
☐	☒	The statistical test(s) used AND whether they are one- or two-sided<br>*Only common tests should be described solely by name; describe more complex techniques in the Methods section.*
☐	☒	A description of all covariates tested
☐	☒	A description of any assumptions or corrections, such as tests of normality and adjustment for multiple comparisons
☐	☒	A full description of the statistical parameters including central tendency (e.g. means) or other basic estimates (e.g. regression coefficient) AND variation (e.g. standard deviation) or associated estimates of uncertainty (e.g. confidence intervals)
☐	☒	For null hypothesis testing, the test statistic (e.g. *F*, *t*, *r*) with confidence intervals, effect sizes, degrees of freedom and *P* value noted<br>*Give P values as exact values whenever suitable.*
☒	☐	For Bayesian analysis, information on the choice of priors and Markov chain Monte Carlo settings
☒	☐	For hierarchical and complex designs, identification of the appropriate level for tests and full reporting of outcomes
☒	☐	Estimates of effect sizes (e.g. Cohen's *d*, Pearson's *r*), indicating how they were calculated

Our web collection on statistics for biologists contains articles on many of the points above.

Software and code

Policy information about availability of computer code

Data collection	Not used
Data analysis	Not used

For manuscripts utilizing custom algorithms or software that are central to the research but not yet described in published literature, software must be made available to editors/reviewers. We strongly encourage code deposition in a community repository (e.g. GitHub). See the Nature Research guidelines for submitting code & software for further information.

Data

Policy information about availability of data

All manuscripts must include a data availability statement. This statement should provide the following information, where applicable:

- Accession codes, unique identifiers, or web links for publicly available datasets
- A list of figures that have associated raw data
- A description of any restrictions on data availability

Accession number GSE117029; Should be publicly available from July 2019.
Reviewer token for access: wlizayokhdujnsj

Field-specific reporting

Please select the one below that is the best fit for your research. If you are not sure, read the appropriate sections before making your selection.

☒ Life sciences ☐ Behavioural & social sciences ☐ Ecological, evolutionary & environmental sciences

Life sciences study design

All studies must disclose on these points even when the disclosure is negative.

Sample size	Sample size was calculated for survival each experiment, targeting an alpha of 0.05, 90% power and and effect size between 25-50%. For flowcytometry, we started with a pilot sample size of 5/group/time point. We replicated the data 3-4 times for all significant results.
Data exclusions	No data was excluded.
Replication	Most experiments were repeated 3 or more times. Initially both genders were included. Since there were no differences in the two genders of weight trajectory, mortality and viral titers, some later experiments were done with males alone. All survival studies however, included both genders.
Randomization	Animals were assigned to treatment groups by simple randomization.
Blinding	All animals were given unique identifiers and the key was accessed only after the images, cell counts or other analyses had been completed.

Reporting for specific materials, systems and methods

We require information from authors about some types of materials, experimental systems and methods used in many studies. Here, indicate whether each material, system or method listed is relevant to your study. If you are not sure if a list item applies to your research, read the appropriate section before selecting a response.

Materials & experimental systems

n/a	Involved in the study
☐	☒ Antibodies
☐	☒ Eukaryotic cell lines
☒	☐ Palaeontology
☐	☒ Animals and other organisms
☒	☐ Human research participants
☒	☐ Clinical data

Methods

n/a	Involved in the study
☒	☐ ChIP-seq
☐	☒ Flow cytometry
☒	☐ MRI-based neuroimaging

Antibodies

Antibodies used

Antibody-Clone- Vendor_ Fluorophore -catalog number -lot number
MHCII M5/114.15.2 eBioscience AF700 56-5321-82 1919519
NK1.1 PK136 Biolegend PerCP-Cy5 108716 B242312
Ly6C HK1.4 Biolegend AF700 128024 B243043
CD11c N418 Biolegend APCCy7 117324 B237079
CD8 53-6.7 Biolegend APCCy7 100714 B237526
Ly-6G 1A8 Biolegend FITC, Pe 127606, 127608 B261239, B258704
CD11b M1/70 eBioscience FITC, APC cy7 11-0112-82, 47-0112-82 4341634, 1950123
CD4 GK1.5 Biolegend FITC 100406 B245891
CD3 17A2 eBioscience FITC 11-0032-82 1959695
Ly-6G 1A8 Biolegend PB, FITC, PE 127606, 127608 B261239, B258704
CD45 30-F11 Biolegend PE/Cy7 103114 B243728, B243728
CD4 GK1.5 Biolegend FITC 100406 B245891
SiglecF E50-2440 BD Pharmingen PE 552126 7208832
CD62L MEL-14 Biolegend PE 104407 B242685
CD103 2E7 eBioscience PerCP-710 46-1031-82 4339659
CD44 IM7 eBioscience PerCP-Cy5.5 45-0441-82 4329935
Fcblock 93 Biolegend N/A 101320 b218499

Validation

Most antibodies had been validates as noted on the manufacturer's website and previous publications. However, prior to each experiment using a new antibody panel, pilot experiment was performed the day before to test all antibodies' performance relevant to the present panel.

Eukaryotic cell lines

Policy information about cell lines

Cell line source(s)

MDCK cell line: originally purchased from ATCC as clarified in the methods.(Used in co-author, Dr. Carolina Lopez's lab to run viral titration as described in methods.)

Authentication	MDCK cell line was authenticated using standard American Type Tissue Collection methods including morphology check by microscope and growth curve analysis.
Mycoplasma contamination	Tested and found to be negative.
Commonly misidentified lines (See ICLAC register)	No commonly misidentified cell lines were used.

Animals and other organisms

Policy information about studies involving animals; ARRIVE guidelines recommended for reporting animal research

Laboratory animals	C57Bl6J were procured from Jackson Labs or bred in house (from breeders obtained from Jackson Labs) for all experiments with wild type mice. For transgenic mice, ER-cre Bmal1flf/fl mice and LysMcre Bmal1fl/fl mice were on a C57bl6 background. the CCsp-cre mice were on a mixed Balbc/C57 background. They have been backcrossed to a C57bl6 background for 6 generations at the time of the experiments. Since Cre- littermates of CCSP mice were used as controls, the background strain was well-controlled for and proves that circadian gating of response to influenza virus seems independent of the background strain. All animals across experiments were between 8-18 weeks of age.
Wild animals	not used
Field-collected samples	not used
Ethics oversight	University of Pennsylvania IACUC

Note that full information on the approval of the study protocol must also be provided in the manuscript.

Flow Cytometry

Plots

Confirm that:

☒ The axis labels state the marker and fluorochrome used (e.g. CD4-FITC).

☒ The axis scales are clearly visible. Include numbers along axes only for bottom left plot of group (a 'group' is an analysis of identical markers).

☒ All plots are contour plots with outliers or pseudocolor plots.

☒ A numerical value for number of cells or percentage (with statistics) is provided.

Methodology

Sample preparation	Lungs were harvested after PBS perfusion through the right ventricle. The lungs were digested using DNAse II (Roche) and Liberase (Roche) at 37oC for 30 mins. Dissociated lung tissue was passed through a 70 um cell strainer, followed by centrifugation and RBC lysis. Cells were washed and re-suspended in PBS with 2% FBS. 2-3 million cells were blocked with 1ug of anti-CD16/32 antibody, washed with FACS buffer and were stained with indicated antibodies on ice for 20-30 minutes. No fixatives were used.
Instrument	Flowcytometric data was acquired using FACS Canto flow cytometer a
Software	Data was analyzed using FlowJo software (Tree Star, Inc.).
Cell population abundance	Sorting was not done.
Gating strategy	All cells were pre-gated on size as singlet live cells. All subsequent gating was on CD45+ in lung only. Neutrophils were identified as live, CD45+, Ly6G+ cells. Ly6Chi monocytes were identified as live, CD45+Ly6G-Ly6ChiCD11b+ cells. NK cells were identified as CD45+Ly6G-LysC-NK1.1+ cells. In some experiments where indicated in the figure legend, an exclusion gate for neutrophils and T, cells (Ly6G, CD4 and CD8) was applied. Alveolar macrophages were identified as CD45+ Ly6G- Siglec F+; DCs were identified as live, CD45+Ly6G-SiglecF-CD11c+MHCII+ cells and further classified into CD103+ conventional DCs or CD11b+ DCs. Day 6 onwards, T cells were identified in mediastinal LNs as CD45+, either CD4+ or CD8+ cells. Activated cells were further differentiated as CD44+, CD62Llo.

☒ Tick this box to confirm that a figure exemplifying the gating strategy is provided in the Supplementary Information.
